# Factors associated with head circumference and indices of cognitive development in early childhood

**DOI:** 10.1136/bmjgh-2020-003427

**Published:** 2020-10-28

**Authors:** Laura Nicolaou, Tahmeed Ahmed, Zulfiqar Ahmed Bhutta, Pascal Bessong, Margaret Kosek, Aldo A M Lima, Sanjaya Shrestha, Ram Chandyo, Estomih R Mduma, Laura Murray-Kolb, Brooks Morgan, Matthew R Grigsby, William Checkley, MALED Network Investigators

**Affiliations:** 1 Division of Pulmonary and Critical Care, School of Medicine, Johns Hopkins University, Baltimore, Maryland, USA; 2 Center for Global Non-Communicable Diseases Research and Training, Johns Hopkins University, Baltimore, United States; 3 Division of Nutrition and Food Security, International Centre for Diarrhoeal Disease Research, Matlab, Bangladesh; 4 Centre for Global Child Health, The Hospital for Sick Children, Toronto, Ontario, Canada; 5 HIV/AIDS and Global Health Research Programme, University of Venda, Thohoyandou, Limpopo, South Africa; 6 Division of Infectious Diseases and International Health, University of Virginia, Charlottesville, Virginia, USA; 7 Clinical Research Unit and Institute of Biomedicine, Faculty of Medicine, Univ Fed Ceara, Fortaleza, Ceará, Brazil; 8 Walter Reed Armed Forces Research Institute of Medical Sciences (AFRIMS) Research Unit, Kathmandu, Nepal; 9 Department of Community Medicine, Kathmandu Medical College, Sinamangal, Kathmandu, Nepal; 10 Haydom Lutheran Hospital, Haydom, United Republic of Tanzania; 11 Department of Nutritional Sciences, College of Health and Human Development, The Pennsylvania State University, University Park, Pennsylvania, USA

**Keywords:** child health, epidemiology, paediatrics

## Abstract

**Background:**

While head circumference (HC) has been related to intracranial volume and brain size, its association with cognitive function remains unclear. We sought to understand the relationship among various biological and socioeconomic risk factors, HC and cognitive development.

**Methods:**

We analysed data across resource-poor settings in Bangladesh, India, Nepal, Peru, South Africa and Tanzania from the Etiology, Risk Factors and Interactions of Enteric Infections and Malnutrition and the Consequences for Child Health and Development longitudinal birth cohort study. Participating children were enrolled and followed up between 2009 and 2014. A final sample of 1210 children aged 0–24 months were included in the analyses. The main outcomes were HC for age Z-score and cognitive, gross motor and language scores from Bayley Scales of Infant Development-III tests. Length, weight and HC were measured monthly, and cognitive tests were administered at 6, 15 and 24 months of age. To disentangle the associations between risk factors and HC from linear growth and to distinguish the direct and indirect effects of these risk factors on cognitive function, we conducted mediation analysis using longitudinal models to account for all data measured during follow-up.

**Results:**

Average HC-for-age Z-score (HCAZ) was −0.54 (95% CI −0.47 to −0.62) near birth and −1.01 (95% CI −0.94 to −1.08) at 24 months. Children with higher enrolment weight (p<0.0001), higher socioeconomic score (p=0.00037) and taller mothers (p=0.00084) had higher HCAZ at all ages, while enteropathogen infection (p=0.013) and more febrile episodes (p=0.013) were associated with lower HCAZ. The associations between HCAZ and enrolment weight-for-age, maternal height, socioeconomic status or pathogen burden were partly mediated through their associations with length-for-age. HCAZ showed no association with cognitive, gross motor or language skills at 6, 15 and 24 months of age.

**Conclusions:**

The main risk factors associated with HC are similar to those associated with body length, and HC is not related to cognitive function.

**Key questions**

**What is already known?**

- Head circumference (HC) is a common anthropometric measurement that is used routinely in paediatric research and surveillance.
- It has been related to intracranial volume and brain size, but its association with cognitive function and the complex relationship between nutritional and environmental factors, body length, HC and cognitive outcomes remain unclear.

**What are the new findings?**

- Mediation analysis allowed us to disentangle associations between risk factors and HC from linear growth and to distinguish the direct and indirect effects of these risk factors on cognitive function.
- The main risk factors affecting HC were found to be similar to those affecting body length.
- We found no association between HC and cognitive function, gross motor function or language skills.

**What do the new findings imply?**

- Our results contribute to a growing body of literature that questions the value of HC as a marker of cognitive function in healthy children.
- These findings add to the limited literature on the relationship between HC and cognition in LMIC settings.

## Introduction

Head circumference (HC) has been long considered a sensitive marker of neurodevelopment.^1 2^ Indeed, the American Academy of Pediatrics recommends measuring HC eight times during the first 2 years of life.^3^ In many settings in low-income and middle-income countries (LMICs) however, HC measurements are not performed regularly.^4^ Furthermore, there is some controversy about the utility of measuring HC for surveillance in healthy children beyond the neonatal period. The United Kingdom Royal College of Paediatrics and Child Health for example currently recommends measuring HC at birth and 8 weeks.^5^

HC during early childhood development is associated with many nutritional factors, including protein energy, micronutrient intake and breastfeeding.^6–8^ Previous studies have examined the association between HC and intracranial volume, tying smaller heads to reduced brain volumes. However, it is unclear if the risk factors associated with HC growth impairment are independent from those associated with linear growth and if HC deficits are associated with cognitive impairments.^9–11^ Indeed, an analysis of 10 851 children found extreme HC measurements to be neither sensitive nor specific in identifying neurocognitive disorders.^9^ Another study of 2104 children from a birth cohort in Spain found no associations between HC or HC growth and mental or psychomotor scores at 14 months of age,^12^ while an analysis of 505 full-term born children in South India found a positive association between HC at birth and subsequent cognitive abilities.^13^ While correlations have been established between nutritional and environmental factors, body length, HC and cognitive outcomes, the complex relationship between these facets remains unclear. Furthermore, there is a dearth of information on the association between HC and cognitive function in LMIC settings.

**Figure 1:**
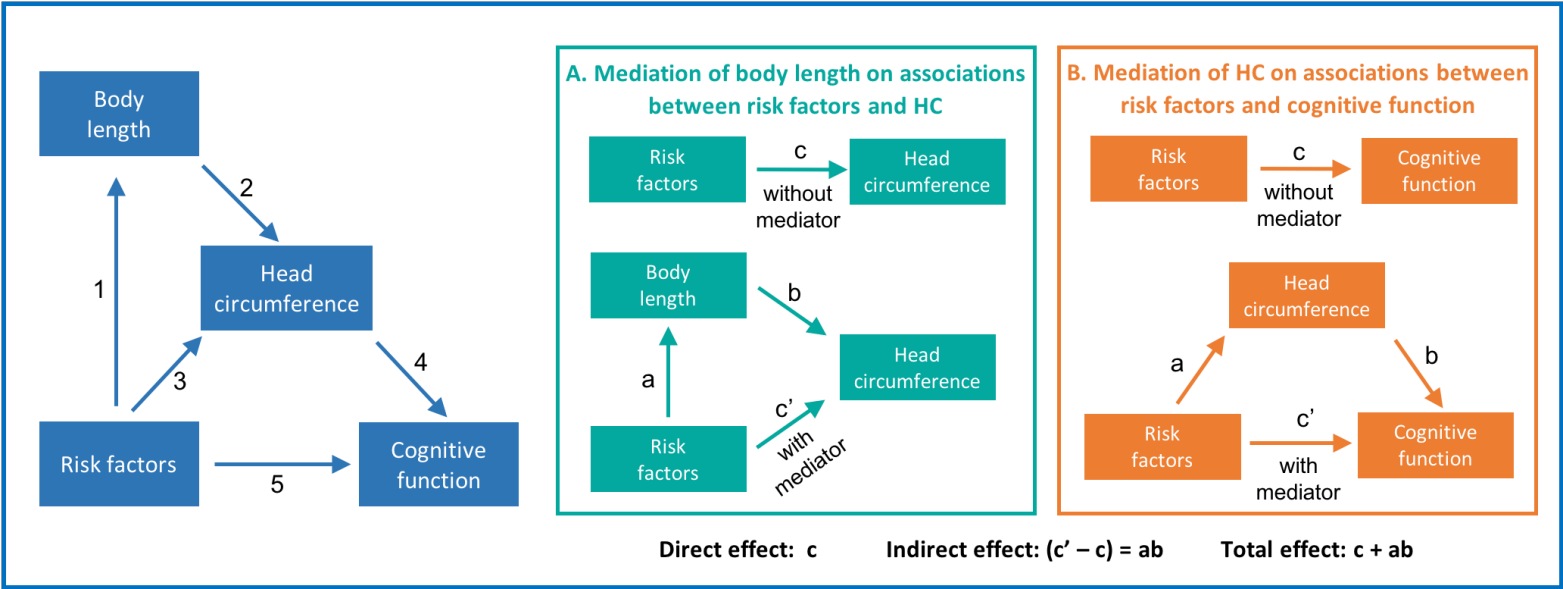
Schematic illustrating the relationships between risk factors, body length, head circumference and cognitive function. Panels A and B are path diagrams of the mediation analyses depicting how the relationbetween risk factors and HC is mediated by body length (A), and how the relation between risk factors and cognitive function is mediated by HC (B). Path a represents the effect of the risk factors on the mediator (body length in A; HC in B). Path b represents the impact of the mediator on our dependent variable (HC in A; cognitive function in B). Together, paths a and b represent the indirect effect of the risk factors on the dependent variable through the mediator. Path c represents the direct effect of the risk factors on HC (A) or cognitive function (B), which is calculated controlling for the indirect effect. Path c represents the total (indirect plus direct) effect of the risk factors on HC (A) or cognitive function (B). HC, head circumference

Since body length, HC and cognitive function are all inter-related,^14–16^ mediation analysis provides a useful framework to disentangle associations between risk factors and HC from linear growth, and to distinguish the direct and indirect effects of these risk factors on cognitive function^17^ (figure 1). We analysed data from a multicentre cohort of children living in eight resource-poor settings of LMICs followed from birth to 24 months. In a previous study,^18^ we determined the associations between risk factors and body length (Pathway 1). Factors that contributed to the odds of being in a lower body length-for-age category at 24 months were lower enrolment weight-for-age, shorter maternal height, higher number of enteropathogens in non-diarrheal stools, lower socioeconomic status and lower per cent of energy from protein. Site-specific analyses suggested that these associations were similar across settings.

Here, we examine the association between body length and HC (Pathway 2), the determinants of HC (Pathway 3) and the associations of cognitive function with HC (Pathway 4) and risk factors (Pathway 5). We also evaluate the indirect effects of body length on the associations between these risk factors and HC (Panel A) and those of HC on the associations with cognitive function (Panel B) to provide new insight into the complex relationship between all these facets. With these analyses, we seek to determine whether HC is a useful marker for cognitive function in healthy children and to establish whether any of our a priori determined risk factors affect HC growth differently from linear growth. Our hypothesis is that the same risk factors that predict body length also predict HC and that HC predicts cognition.

## Methods

### Study design and participants

We analysed data from the Etiology, Risk Factors and Interactions of Enteric Infections and Malnutrition and the Consequences for Child Health and Development (MAL-ED) Network cohort study, a multidisciplinary, prospective, longitudinal study aimed at better understanding the complex relationships among enteric infections, nutrition and other environmental exposures on child growth and development. Participating children were enrolled and followed up between 2009 and 2014 at eight field sites: Dhaka, Bangladesh (BGD); Fortaleza, Brazil (BRF); Vellore, India (INV); Bhaktapur, Nepal (NEB); Naushahro Feroze, Pakistan (PKN); Loreto, Peru (PEL); Venda, South Africa (SAV) and Haydom, Tanzania (TZH).^19^ After a baseline community census, enrolment began with the goal of recruiting 200 children per site. All infants were enrolled within 17 days of birth and followed continuously until 24 months.

### Participant and public involvement

Neither participants nor the public were involved in the design, conduct or reporting of our study.

### Anthropometric measurements and cognitive function

The main outcome for this analysis was HC for age Z-score (HCAZ) and cognitive, gross motor and language scores from the Bayley Scales of Infant Development-III (BSID-III) tests. We measured length, weight and HC in children monthly from the first 2 weeks of life. Several quality control measures were implemented, including the use of standardised techniques and instruments across all sites, same-day review of growth curves to identify unlikely measurements and duplicate anthropometric measurements on a subset of children.^20^ We standardised all anthropometric measurements into Z-scores using the WHO growth standard.^21^

We assessed cognitive development through periodic administration of the BSID-III test for cognitive skills and gross motor function at 6, 15 and 24 months and language development at 15 and 24 months. At every site, the BSID-III test was translated, adapted to local culture and piloted on a separate group of children to ensure comparable difficulty of each item to the original item.^22^ Psychometric analyses were completed, and items with sufficient variability were kept (range 0–15). We excluded TZH from the cognitive development analyses, as quality control reviews revealed that the administration of cognitive tests deviated significantly from the protocol.^23^ Cognitive scores from NEB at the 24-month time point were also excluded, as psychometric analyses suggested that the questions performed different compared with the other sites.^23^

### Biological and socioeconomic factors

Risk factors considered were weight-for-age Z-score at enrolment, total energy intake in kilocalories, cumulative proportion of days that a child was breastfed, micronutrient levels (inflammation-adjusted ferritin and retinol), altitude-adjusted haemoglobin concentration, maternal height, socioeconomic status (mean water and sanitation, wealth and assets, maternal education and household income (WAMI) score), food insecurity, diarrheal episodes, longitudinal prevalence of fever, acute lung respiratory infection (ALRI), antibiotic use, faecal pathogen burden, alpha-1-acid glycoprotein (AGP) plasma concentration, myeloperoxidase (MPO), neopterin (NEO) and alpha-1 antitrypsin (AAT) concentrations, and lactulose:mannitol Z-score.

The methods of data collection for feeding, illness surveillance and microbiology are described elsewhere.^24–29^ Briefly, teams of field workers visited the households of participating children two times a week to attain daily reports of childhood illness (diarrhoea, acute lower respiratory infection and fever) and antibiotic use. Longitudinal prevalence was calculated as the number of days ill (or with antibiotic use) divided by the number of follow-up days.^18^ To assess micronutrient status, systemic inflammation, enteropathogen burden and gut inflammation and permeability markers, longitudinal visits were conducted to collect blood (at 7, 15 and 24 months), urine (at 3, 6, 9 and 15 months) and non-diarrheal stool samples (monthly during the first year of life and quarterly during the second year of life).^19^

From blood, we evaluated concentrations of altitude-adjusted haemoglobin, inflammation-adjusted retinol, AGP as a marker of systemic inflammation and inflammation-adjusted plasma ferritin by averaging the three measurements obtained during the study period. From non-diarrheal stools, we measured gut inflammation biomarkers, including MPO, NEO and AAT concentrations. We calculated quarterly averages from the monthly measurements collected during the first year and used the collected quarterly measurements thereafter.^18^ We computed enteropathogen load as the average number of enteropathogens in non-diarrheal stool divided by the total number of stool samples tested and determined cumulative enteropathogen load, assuming it was zero at enrolment and carrying forward cumulative values for months with missing data. From urine, we evaluated the lactulose:mannitol ratio as a marker of gut permeability and used the value measured at 3 months for the interval birth to 5 months, the 6-month value for the interval 6–8 months, the 9-month value for the interval 9–14 months and the 15-month value for the interval 15–24 months^18 27^. We then transformed the lactulose:mannitol ratios to Z-scores using the BRF data as our internal reference standard. The BRF site had no deviation in anthropometry from the WHO growth standards and was therefore chosen to represent the least abnormal gut permeability values.^28 29^

Initiation of breastfeeding was recorded and surveillance of feeding practices was conducted two times a week. We assumed days between visits to have the same status as the preceding visit^30^ and calculated the cumulative proportion of days that a child was breastfed. Additionally, once a month beginning at 9 months, we conducted 24 hours complementary food intake recall interviews and computed cumulative energy intake from non-breast-milk foods over time, by linking the information provided in the interviews to country-specific nutritional databases. While a single recall does not provide a highly precise estimate of usual energy intake at any given time point due to within-subject (ie, day-to-day) variation, the 16 measures available for each child from 9 to 24 months, can be combined to capture more precise intake information over time. To enhance the data collected, each child was randomly allocated to have a secondary recall done 2–7 days after one of the study visits between 9 and 24 months. This information was used to refine both group and individual estimates of energy intakes beyond the precision afforded by the monthly energy intake estimates.^24^

Socioeconomic status (SES) was quantified using an index combining WAMI index.^31^ Every 6 months, we interviewed mothers or caregivers about their sources of water and sanitation facilities, assets, income and food security. We collected level of maternal education at baseline. As the WAMI index and food insecurity score showed small variation over time, we averaged their values across all collected time points.

### Biostatistical methods

The analytical objectives were to study the association of HC growth with body length (Pathway 2, figure 1) and various biological and socioeconomic risk factors (Pathway 3); determine if the associations between these risk factors and HC were mediated by body length (Panel A); examine the association of cognitive function with HC (Pathway 4) and risk factors (Pathway 5) and last, determine if the associations between these risk factors and cognitive function were mediated by HC (Panel B).

To study the association of HC growth with body length (Pathway 2, figure 1) and various biological and socioeconomic factors (Pathway 3, figure 1), we used linear mixed-effects regression to model longitudinal HCAZ as a function of age, sex, indicator variables for sites and all of the above-mentioned biological and socioeconomic risk factors. All factors except site and sex were included as continuous variables. We modelled age using a piecewise linear spline with knots at age 3, 6 and 12 months and included interactions of the spline elements for age with all risk factors, site and sex. We included random effects at the individual level, accounting for heterogeneity in HC growth and HC at enrolment with random slopes and intercepts, respectively, and adjusted by site as a fixed effect. We built a fully adjusted model which included all the a priori risk factors as well as unadjusted models for each individual biological and socioeconomic risk factor, which included age, and interactions of the spline elements for age with that particular risk factor. We verified goodness-of-fit of the fully-adjusted model by comparing predicted and observed HCAZ trajectories with age (online supplemental efigure 1). Normality of residuals was confirmed through visual inspection (QQ plot of residuals, histogram of residuals, plot of residual versus fitted values).

10.1136/bmjgh-2020-003427.supp1 Supplementary data

To assess the contribution of linear growth on the relationship between risk factors and HC growth (Panel A), we estimated average direct effects (ADE), average causal mediation effects (ACME) and average total effects (ATE) for each risk factor following the methods described by Imai *et al*.^32^ Specifically, we constructed a linear mixed-effects model for HCAZ as a function of above-mentioned variables plus length-for-age Z-score (mediator) and a linear mixed-effects model for length-for-age Z-score (LAZ) as a function of the same variables. We conducted 1000 Monte Carlo simulations to estimate the probability distributions of ADE, ACME and ATE and used a quasi-Bayesian approach to estimate mean and 95% CI.^32 33^ This method also allows for calculation of the percentage of the total association attributed to the mediator.

To evaluate Pathway 4, we modelled the indices of cognitive development at the ages of test administration (cognitive skills and gross motor function at 6, 15 and 24 months and language development at 15 and 24 months) as a function of HC and above-mentioned biological and socioeconomic factors. Since we have a single observation per participant at each time point, we adopted simple linear regression models. We used two types of HC measures: HCAZ and the slope of HCAZ (as a measure of HC growth) at the time of test administration. We estimated individual child slopes for HCAZ using a linear model of HCAZ as a function of age in the intervals between test administrations (0–6 months, 6–15 months and 15–24 months). To capture the non-linear relationships of the two HC measures with cognitive, gross motor and language scores (online supplemental efigure 2), we used truncated cubic regression splines. We also examined the relationship between cognitive function and time-lagged values of HCAZ using piecewise linear splines with knots at the 10th percentile, median and 90th percentile. The models included current (0 lag) and monthly lagged HCAZ in the interval between test administrations, that is, 0–6 months lagged HCAZ at 6 months of age and 0–8 months lagged HCAZ at 15 and 24 months of age.

Finally, using our linear models for the indices of cognitive development as a function of the above-mentioned factors plus HCAZ, and a linear model for HCAZ as a function of the same factors, we conducted mediation analyses as previously described. These analyses allowed us to evaluate the direct effects of these risk factors on cognitive function (Pathway 5) and to determine whether any of the three HC measures mediated the association between the risk factors and cognitive development (Panel B).

We conducted statistical analyses in R V.3.6.2 (Dark and Stormy Night) (http://www.r-project.org).

## Results

### Study population

A total of 2145 children were included in the initial study population. Of those enrolled, 17.1% (n=367) were lost to follow-up and 9.8% (n=210) did not meet minimal criteria^3^ for longitudinal follow-up. Of those with complete data, participants from Pakistan (n=277) were excluded due to bias identified in a subset of length measurements, and participants from Brazil (n=81) were excluded due to bias in the HC measurements. A final sample of 1210 was included in the analyses. Those excluded from analysis varied from those included except in sex and mean WAMI score (online supplemental etable 2). Among those who were included, all baseline features except for sex varied by site (online supplemental etable 3).

### Longitudinal trends in HC

**Figure 2:**
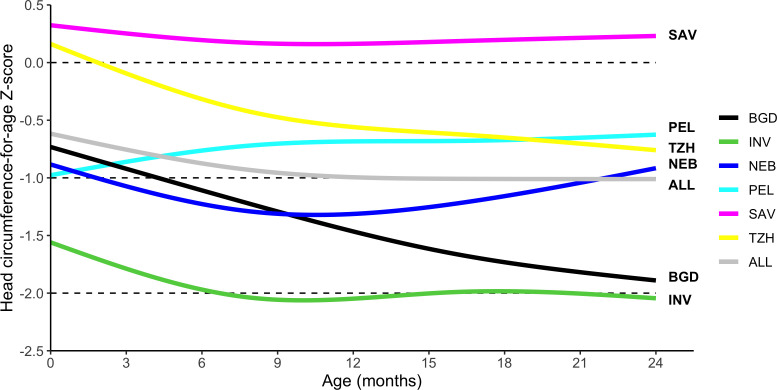
Head circumference-for-age Z-score stratified by age and site. Site-specific head circumference-for-age trajectories with age were smoothed using natural splines. Sites include BGD, Dhaka, Bangladesh; INV, Vellore, India; NEB, Bhaktapur, Nepal; PEL, Loreto, Peru; SAV, Venda, South Africa; TZH, Haydom, Tanzania.

**Figure 3:**
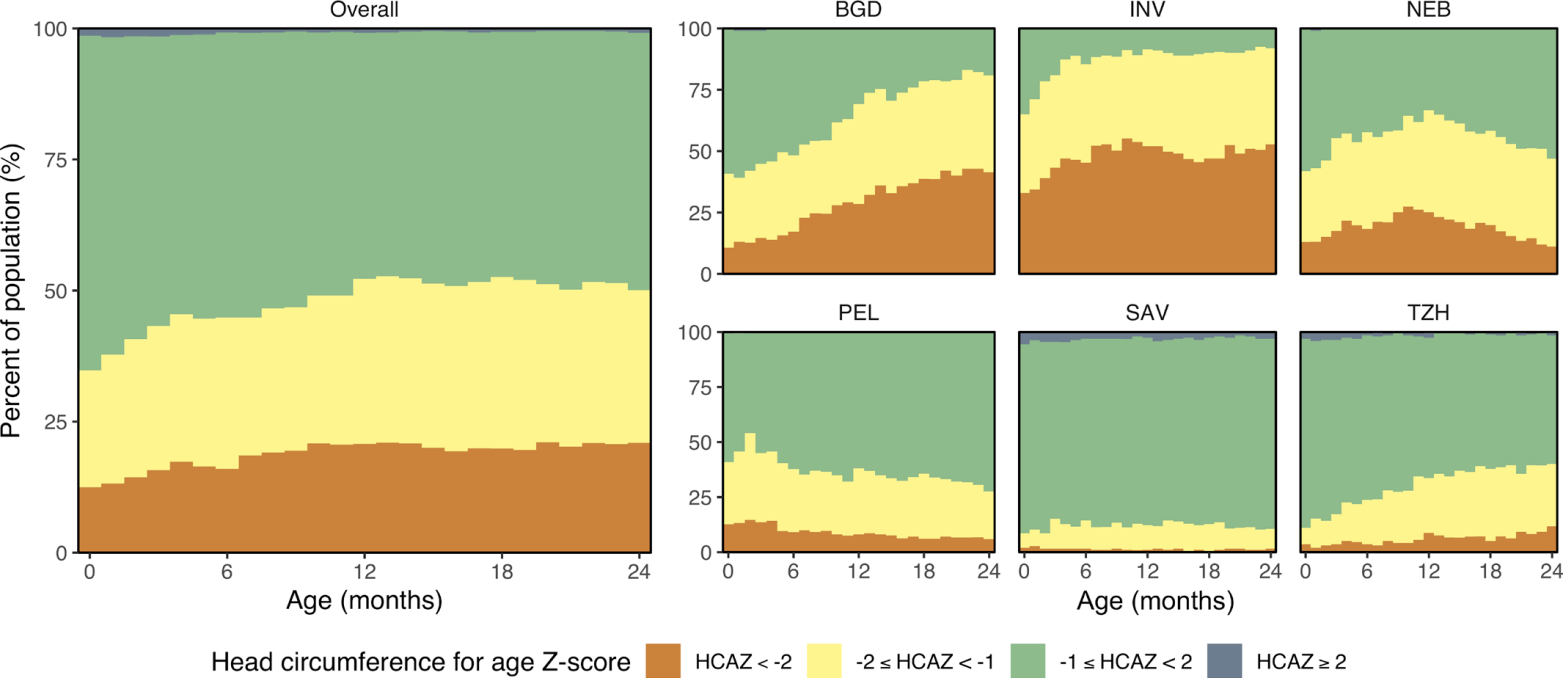
Categories of head circumference-for-age stratified by age and country. Head circumference-for-age Z-score HCAZ<−2 is represented in orange, −2≤HCAZ<−1 is represented in yellow, -1≤HCAZ<2 is represented in green and HCAZ≥2 in dark grey. Sites include BGD, Dhaka, Bangladesh; INV, Vellore, India; NEB, Bhaktapur, Nepal; PEL, Loreto, Peru; SAV, Venda, South Africa; TZH, Haydom, Tanzania.

On average, HCAZ fell with age during the first 9–12 months and remained constant thereafter, decreasing from −0.54 (95% CI −0.47 to −0.62) at enrolment to −1.01 (95% CI −0.94 to −1.08) at 24 months. Site-specific trajectories showed a decrease in HCAZ with age, except for PEL that showed an increase in the first 9 months of life followed by a plateau (figure 2). Mean HCAZs remained below the WHO standard (Z-score <0) across all sites except SAV. At enrolment, 12.6% (n=153) of children (range of 2.0% in SAV to 33.5% in INV) had HCAZ<−2, which rose to 20.8% (n=252) of children at 24 months (range of 1.5% in SAV to 52.5% in INV) (figure 3). Less than 1.5% of all children had HCAZ ≥2.

### Association between body length and HC (Pathway 2)

In online supplemental efigure 3, we show the 95% confidence ellipses for the scatter plots between HCAZ and LAZ by site at 3, 6, 12 and 24 months of age. These confidence ellipses represent the 2D analogue of a CI for bivariate data, with their angle determined by the covariance of the data and the magnitude of the axes determined by the variance. As expected, there was a strong positive correlation between HCAZ and LAZ at all sites and ages. A larger variability in HCAZ compared with LAZ was observed across sites. On the other hand, LAZ displayed a larger decrease with age (mean and 95% CI across all sites: −1.04 (-0.98 to –1.10) at 3 months; −1.91 (−1.85 to −1.97) at 24 months) while HCAZ remained fairly constant beyond 6 months of age in all sites except BGD (mean and 95% CI across all sites: −0.81 (-0.74 to –0.88) at 3 months; −1.01 (−0.94 to −1.08) at 24 months).

### Determinants of HC (Pathway 3)

Overall, a child had a higher HCAZ if he or she had a higher enrolment weight-for-age, was born to a taller mother or lived in a household with a higher WAMI. Pathogen burden and longitudinal prevalence of fever were negatively associated with HC (online supplemental efigure 4). The associations with WAMI index and pathogen burden were greater with age, while the associations with enrolment weight, maternal height and fever weakened with age. HCAZ was also weakly associated with higher energy intake, larger proportion of days breastfeeding, lower prevalence of ALRIs and lower AAT and ferritin concentrations. No associations were found between HCAZ and sex (p=0.49; likelihood ratio test using a χ² distribution with 5 df ($LRTwith\chi_{df=5}^{2}$), haemoglobin (p=0.25; $LRTwith\chi_{df=5}^{2}$), retinol (p=0.23; $LRTwith\chi_{df=5}^{2}$), food insecurity (p=0.13; $LRTwith\chi_{df=5}^{2}$), NEO (p=0.68; $LRTwith\chi_{df=5}^{2}$), MPO (p=0.71; $LRTwith\chi_{df=5}^{2}$), lactulose:mannitol Z-score (p=0.31; $LRTwith\chi_{df=5}^{2}$), AGP (p=0.054; $LRTwith\chi_{df=5}^{2}$), prevalence of diarrheal episodes (p=0.62; $LRTwith\chi_{df=5}^{2}$) or antibiotic use (p=0.054; $LRTwith\chi_{df=5}^{2}$). There was substantial heterogeneity in the associations between risk factors and HCs across sites, with estimated mean differences in HCAZ between the lowest and higher quartiles of these risk factors varying in both magnitude and direction (online supplemental efigure 5).

### Mediation of body length on associations between risk factors and HC (Panel A)

**Figure 4:**
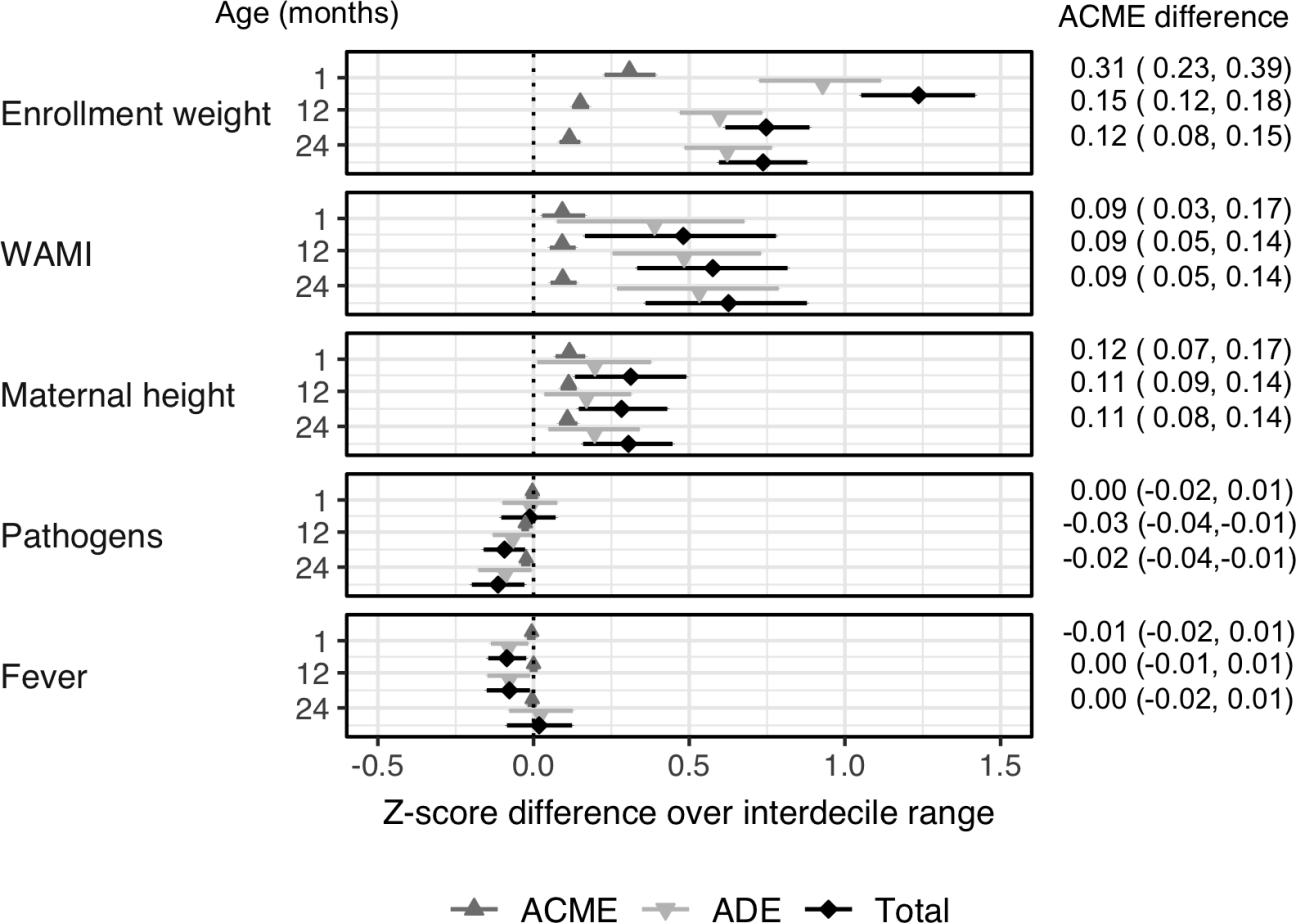
Mediation of body length on head circumference growth during early life development. The symbols represent the direct (ADE), length-mediated (ACME) and total effects on head circumference scaled to the interdecile range of various risk factors at enrolment, 12 months and 24 months of age. For example, the total effect of enrolment weight Z-score (WEIZ) on HCAZ at 1 month of age is 0.456. This value reflects how much one unit change in WEIZ affects HCAZ. To determine how much a difference between the 90% and 10% percentile of WEIZ affects HCAZ, we scale this per-unit effect by the IDR of WEIZ. The 10% and 90% percentiles of WEIZ at 1 month of age are –2.19 and 0.52, respectively; the IDR is 2.71. The total effect over the interdecile range is therefore 1.24 (0.456×2.71) as plotted in the figure. Error bars represent 95% CIs. ACME, average causal mediation effect; ADE, average direct effect; IDR, interdecile range; WAMI, water and sanitation, wealth and assets, maternal education and household income.

The effects of enrolment weight, WAMI index, maternal height and pathogen burden on HCAZ were all mediated by LAZ (figure 4). The indirect effect of body length on the association between maternal height and HCAZ remained fairly constant throughout the first 2 years of life with mediation effects constituting 35%–40% of the total effects. On the other hand, the contribution of mediation effects to the total effects of enrolment weight, mean WAMI index or pathogen score on HCAZ decreased with age. Body length accounted for 25% of the total effect of enrolment weight on HCAZ in the first month of age, dropping to 20% at 12 months and 16% at 24 months. The mediation effect of body length contributed to 19%, 16% and 15% of the total effect of WAMI index on HCAZ at 1, 12 and 24 months, respectively, and 28% and 21% of the total effect of pathogen burden at 12 and 24 months, respectively. No mediation was found on the effects of fever.

### Associations between HC and cognitive function (Pathway 4)

We found no associations between cognitive, gross motor or language scores and any of the three HC measures as assessed at 6, 15 and 24 months of age (online supplemental efigure 6). Furthermore, taking into account various time lags between cognitive function and HCAZ, the vast majority of differences in all three cognitive measures were not significant, and those that were statistically significant, such as the differences in cognitive score at 6 months of age with 0 and 1 month lagged HCAZ, appeared in opposite directions showing no clear association between cognitive function and HCAZ (online supplemental efigures 7 and 8).

### Risk factors for cognitive impairment (Pathway 5) and mediation by HC (Panel B)

**Figure 5:**
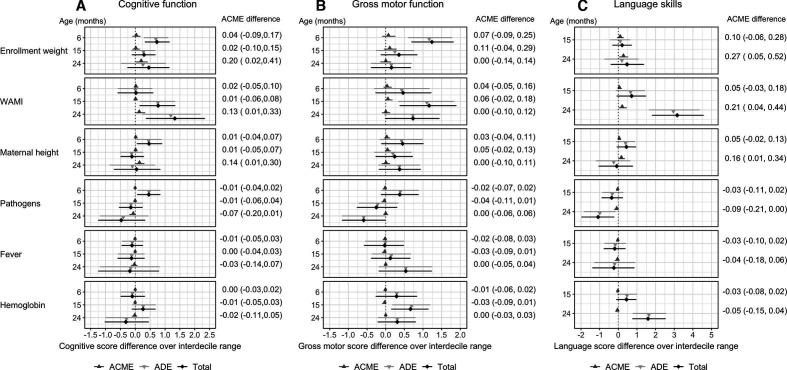
Mediation of head circumference on (A) cognitive function, (B) gross motor function and (C) language skills during early life development. The symbols represent the direct (ADE), head circumference mediated (ACME) and total effects on cognitive, gross motor and language scores over the interdecile range of various risk factors at 6, 15 and 24 months of age. Error bars represent 95% CIs. ACME, average causal mediation effect; ADE, average direct effect.

Cognitive function was positively associated with enrolment weight, maternal height and WAMI (figure 5A). The total effects (ATEs) of WAMI on cognitive score increased with age, while those of enrolment weight and maternal height were only significant at 6 months. We also found negative associations with MPO and AGP concentrations at 15 months of age (online supplemental efigure 9). However, none of these associations were mediated by HC. Higher gross motor function scores were associated with higher enrolment weight, WAMI index and haemoglobin levels (figure 5B). None of these associations were mediated by HC. Associations between gross motor function and all other risk factors were found to be insignificant (online supplemental efigure 10). For language, we found a positive association with WAMI and haemoglobin, and a negative association with pathogen burden (figure 5C). A small positive effect was also observed for lactulose:mannitol at 15 months (online supplemental efigure 11). The causal mediation effect of HC on these associations remained non-significant. Additional mediation analyses conducted using the HCAZ slope and HCAZ/LAZ as HC measures revealed similar results (not presented).

## Discussion

In this analysis, we found that the previously identified biological and socioeconomic factors associated with linear growth^18^ are also associated with HC growth. Moreover, the effects of enrolment weight-for-age, maternal height, WAMI index and pathogen burden on HC were partly mediated through body length. HC was not associated with indicators of cognitive development at any age, whether it was language, cognitive skills or gross motor function; and, HC did not mediate the association between any of the studied risk factors and cognitive development. These findings suggest that the routine measurement of HC may not provide useful information regarding cognitive development in healthy children. Our study also adds to the limited literature on the relationship between HC and cognition in LMIC settings.

Our findings are consistent with those observed by other investigators in different settings.^9–12^ Indeed, the Avon Longitudinal Study of Parents and Children (ALSPAC) study found that most children with small heads will have no underlying pathology and that the most common explanation for centile shifts in HC is measurement error.^9^ ALSPAC investigators concluded that longitudinal HC assessments were unhelpful (unless <2 SDs or >2 SDs below or above the median) and that HC surveillance should be conducted at 2–5 days after birth and then at 6 months of age. Treit *et al* studied 144 children with prenatal alcohol exposure and 145 healthy children without prenatal alcohol exposure but did not find significant associations between HC and any cognitive score and called into question the predictive value of HC at the individual-subject level.^10^ Our findings provide further support to the notion that longitudinal assessment of HC is neither helpful nor cost-effective for research or surveillance in healthy children.^9 12 34^ One possible exception could be screening for hydrocephalus. A population-based study conducted in Norway found that increased HC as a debut symptom was only important in children with hydrocephalus and cysts, but only during the first 10 months of life.^35^ The authors concluded that the benefits of extending routine measurements of HC even for hydrocephalus beyond 1 year of age is questionable. Another study conducted in Netherlands found the same results.^36^

Overall, we observed similar trends of a fall in HC with age and similar relationships between risk factors and HC as in a previous analysis conducted by our group where linear growth was the main outcome.^18^ HC decreased on average between birth and the first years of life at roughly the same magnitude as it did for body length. The risk factors for a lower HC gain were also similar to those identified for linear growth, namely, a lower enrolment weight, lower maternal height, lower socioeconomic status and a higher prevalence of enteric pathogens. Finally, the direction and magnitude of association of these risk factors with age on HC were similar to those documented for body length. Mediation analyses revealed that the effects between risk factors and a lower HC were explained in part by their contributions to a lower body length. Our findings are also consistent with a single-country analysis of data collected in India,^5^ where our colleagues found that HC was negatively associated with stunting at all ages and that a lower socioeconomic status was associated with a lower HC. Our analyses and findings differ from those presented in a prior study^37^ which focused on the association between HC and cognitive function adjusted only for enrolment weight and site and point towards a potential residual confounding in the results presented by Scharf *et al*.^37^ One concern about our analysis is the potential overfitting given the large number of model parameters; however, our results were unchanged whether we adjusted for the five most important risk factors or for the entire set of risk factors.

An important secondary finding of this analysis was the relationship between a lower socioeconomic status and worse cognitive development in our study children. This finding is not entirely surprising as the association between childhood socioeconomic status and late-life cognition in the US, Europe and China is well documented.^38–41^ While the mechanisms are not well understood, our findings demonstrate that factors which predict linear growth and HC do not similarly predict cognition, which suggests that socioeconomic adversity affects brain development through its effects on diet and other non-nutritional factors.^41^ There may also be other nutrient deficiencies not considered in this study that could be related to cognition even though factors that predict growth and head size might not similarly predict cognition. Mechanisms notwithstanding, this finding suggests that one of the most important interventions that can lead to improvements in child growth and development is poverty reduction. Moreover, our analysis adds evidence on the importance of life-course socioeconomic status on cognitive development disparities.

Our analysis has several strengths. First, the MAL-ED study collected a comprehensive assortment of prenatal and postnatal factors across a wide array of settings worldwide, each of which used a standardised protocol and data collection toolset. As this was a prospective study, repeat visits allowed for accurate collection of pathogen, nutrition and illness surveillance data over time. Second, our study conducted high-quality anthropometric assessment which allowed us to examine monthly changes in body length and HC early in childhood and relate it to several biological and socioeconomic risk factors. Third, our analyses used rigorous statistical methods to help disentangle the relationships between risk factors, body length, HC and cognition. These analyses were conducted on random samples of infants in resource-limited settings across six different LMICs. Future studies in other countries should determine whether our findings are generalisable to other settings.

This analysis also has some potential shortcomings. First, the relationships between genetic, nutritional, socioeconomic and environmental factors are complex and our results may include unaccounted for confounding or feedback effects. Second, although the MALED study contributed data from eight sites, all data from Pakistan had to be dropped due to bias in length measurements, all data from Brazil had to be dropped due to bias in HC measurements and all cognitive data from Tanzania had to be dropped due to bias in the cognitive development assessment. Third, our study failed to collect information on gestational age. While our eligibility criteria excluded those with a birth weight <1.5 kg, this criterion may not adequately screen for preterm birth or severe intrauterine growth restriction. We should also note that our analysis did not account for the role of caregiving and early-life stimulation and play in cognitive development. Other important limitations that need to be considered are the loss of higher-risk infants from study follow-up; limited predictive value of cognitive testing at 24 months for school-age children; lack of validation for the Bayley scales in our study settings and the unknown predictive value of the newly created subscale. However, adaptation, piloting and validity assessments of the BSID-III tests were conducted at each of the study sites.^22^

## Conclusion

In conclusion, using data from one of the most comprehensive longitudinal studies of child growth and development in LMICs, and accounting for the most extensive array of confounders to date, we found that the main risk factors associated with HC were similar to those associated with body length. We did not find an association between HC and cognitive function and did not find an indirect effect of HC on the association between cognitive function and other risk factors.
