# Supplementary material for: Factors associated with head circumference and indices of cognitive development in early childhood
Source: BMJ Glob Health. 2020 Oct 28;5(10):e003427. doi: 10.1136/bmjgh-2020-003427 (PMC7594357; doi:10.1136/bmjgh-2020-003427)
Supplement: Supplementary data [file bmjgh-2020-003427supp001.pdf]

Supplementary Material

Factors associated with head circumference and indices of cognitive development in early childhood

eTable 1. Institutional Research Boards Approvals.

eTable 2. Comparison of participants included vs. excluded from analysis. P value represents results from t-tests.

eTable 3. Comparison of field sites by baseline features. P value represents results from ANOVA tests.

eFigure 1. Goodness of fit. Mean and 95% confidence intervals of predicted and observed site-specific and overall head circumference-for-age trajectories with age. Predicted values as obtained from the multivariable linear mixed effects model.

eFigure 2. **Cognitive, gross motor and language scores as a function of head circumference for age Z-score (HCAZ), HCAZ slope and ratio of HCAZ to length for age Z-score (HCAZ/LAZ) stratified by site.**

eFigure 3. Head circumference-for-age Z- score vs length-for-age Z-score stratified by site. The ellipses represent the 95% confidence regions for the scatterplot between head circumference-for-age Z- score and length-for-age Z-score each site at 3, 6, 12, and 24 months of age.

eFigure 4. **Overall change in head circumference for age Z-score (HCAZ) at enrollment, 12, and 24 months of age for all risk factors considered.** Estimated mean differences in HCAZ are plotted over ranges in the risk factors between the second, third or fourth quartiles and the first quartile, as obtained from the unadjusted and adjusted linear mixed effects models.

eFigure 5. **Change in head circumference for age Z-score (HCAZ) by site at enrollment, 12, and 24 months of age for all risk factors considered.** Estimated mean differences in HCAZ are plotted over ranges in the risk factors between the second, third or fourth quartiles and the first quartile, as obtained from the unadjusted and adjusted linear mixed effects models.

eFigure 6. **Change in cognitive, gross motor and language scores with head circumference for age Z-score (HCAZ), HCAZ slope and ratio of HCAZ to length for age Z-score (HCAZ/LAZ) at 6, 15, and 24 months of age.** Estimated mean differences are plotted over the quartile increases, Q2-Q1 and Q3-Q2, of HCAZ, HCAZ slope and HCAZ/LAZ as obtained from the unadjusted models and the models adjusted for enrollment weight and site, adjusted for the main factors (enrollment weight, site, HCAZ, maternal height, mean WAMI, mean hemoglobin concentration, pathogen burden, fever episodes), and adjusted for all factors.

eFigure 7. Change in cognitive, gross motor and language scores with lagged head circumference for age Z-score (HCAZ) at 6, 15, and 24 months of age. Estimated mean differences in the BSID-III test scores are plotted over one standard deviation below the median of HCAZ, taking into account various lags between cognitive function and HCAZ, as obtained from models adjusted for all factors.

eFigure 8. Change in cognitive, gross motor and language scores with lagged head circumference for age Z-score (HCAZ) at 6, 15, and 24 months of age. Estimated mean differences in the BSID-III test scores are plotted over one standard deviation above the median of HCAZ, taking into account various lags between cognitive function and HCAZ, as obtained from models adjusted for all factors.

eFigure 9. Mediation of head circumference on cognitive function during early life development. The symbols represent the direct (ADE), head circumference-mediated (ACME) and total effects on cognitive score over the interdecile range of all risk factors considered at 6, 15 and 24 months of age. Error bars represent 95% confidence intervals.

eFigure 10. Mediation of head circumference on gross motor function during early life development. The symbols represent the direct (ADE), head circumference-mediated (ACME) and total effects of various risk factors on gross motor function at 6, 15 and 24 months of age. Error bars represent 95% confidence intervals.

eFigure 11. Mediation of head circumference on language skills during early life development. The symbols represent the direct (ADE), head circumference-mediated (ACME) and total effects of various risk factors on language skills at 15 and 24 months of age. Error bars represent 95% confidence intervals.

eTable 1. Institutional Research Boards approvals.

**Nepal**	
	Institute of Medicine, TU; Institutional Review Board
	Nepal Health Research Council; Ethical Review Board
	Walter Reed Army Institute of Research; Institutional Review Board
**Pakistan**	
	Aga Khan University; Ethical Review Committee
**Peru**	
	Bloomberg School of Public Health, Johns Hopkins University; Institutional Review Board
	A.B. PRISMA Ethics Committee
	Health Ministry, Loreto
**South Africa**	
	University of Venda; Health, Safety and Research Ethics Committee
	Limpopo Provincial Government; Dept of Health and Social Development
	University of Virginia; Institutional Review Board for Health Sciences Research
**Tanzania**	
	National Institute for Medical Research; Medical Research Coordinating Committee
	Ministry of Health and Social Welfare; Chief Medical Officer
	University of Virginia; Institutional Review Board for Health Sciences Research
**India**	
	Christian Medical College; Institutional Review Board
	Indian Council of Medical Research; Health Ministry Screening Committee
**Brazil**	
	Universidade Federal do Ceara; Committee for Ethics in Research
	Health Ministry, Council of National Health; National Ethical Research Committee
	University of Virginia; Institutional Review Board for Health Sciences Research
**Bangladesh**	
	ICDDR,B; Ethical Review Committee
	University of Virginia; Institutional Review Board for Health Sciences Research

eTable 2. Comparison of participants included vs. excluded from analysis. P value represents results from t-tests.

	**Included** (77.2%, n=1,210)	**Excluded** (22.8%, n=358)	**P value**
**Demographics:** % (n) or median (IQR)			
Female Sex	48.9% (592)	48.0% (172)	0.77
Weight at Enrollment (Z-score)	-0.67 (-1.42, -0.05)	-1.14 (-1.86, -0.47)	<0.0001
Maternal Height (cm)	152 (147.5, 156.7)	153.2 (149.5, 157.2)	0.00022
Maternal Education (≥6 years)	67.8% (774)	32.5% (112)	<0.0001
Income >150 USD/month	29.3% (334)	58.0% (200)	<0.0001
Inadequate Sanitation	38.3% (437)	17.7% (61)	<0.0001
Inadequate Water	16.0% (182)	0% (0)	<0.0001
WAMI Score	0.56 (0.39, 0.70)	0.56 (0.38, 0.77)	0.050
Mean Food Insecurity Score	1.2 (0, 4.5)	5.6 (2.2, 8.8)	<0.0001

Nicolaou L, *et al. BMJ Global Health* 2020; 5:e003427. doi: 10.1136/bmjgh-2020-003427

eTable 3. Comparison of field sites by baseline features. P value represents results from ANOVA tests.

	**Bangladesh** (16.0%, n=206)	**India** (15.5%, n=200)	**Nepal** (16.1%, n=208)	**Peru** (15.3%, n=198)	**South Africa** (15.4%, n=199)	**Tanzania** (15.4%, n=199)	**P value**
**Demographics:** % (n) or median (IQR)							
Female Sex	49.0% (101)	55.5% (111)	46.2% (96)	43.5% (86)	49.2% (98)	50.2% (100)	0.22
Weight at Enrollment (Z-score)	-1.29 (-1.89,0.58)	-1.19 (-1.88, -0.52)	-0.76 (-1.37, -0.27)	-0.61 (-1.16, -0.05)	-0.29 (-0.93, 0.29)	-0.11 (-0.61, 0.52)	<0.0001
Maternal Height (cm)	149.2 (145.7, 152.5)	151.1 (148.0, 154.5)*	149.6 (146.4, 152.7)	149.0 (146.0, 153.5)	159.1 (155.0, 162.6)*	156.2 (152.0, 159.4)	<0.0001
Maternal Education (≥6 years)	36.5% (70)*	63.8% (127)*	74.0% (154)	77.0% (151)*	98.0% (148)*	63.6% (124)*	<0.0001
Income >150 USD/month	24.5% (47)*	6.0% (12)*	46.2% (96)	42.9% (84)*	59.6% (90)*	2.6% (5)*	<0.0001
Inadequate Sanitation	0% (0)*	54.3% (108)*	0% (0)	74.5% (146)*	3.3% (5)*	91.3% (178)*	<0.0001
Inadequate Water	0% (0)*	0% (0)*	0.5% (1)	8.2% (16)*	21.2% (32)*	68.2% (133)*	<0.0001
WAMI Score	0.53 (0.45, 0.63)	0.48 (0.36, 0.57)	0.69 (0.62, 0.80)	0.54 (0.46, 0.62)	0.79 (0.72, 0.85)	0.21 (0.14, 0.28)	<0.0001
Mean Food Insecurity Score	0 (0, 1.6)	0 (0, 2.0)	0 (0, 1)	6.4 (4.4, 9.8)	3 (0.8, 6.23)	1.2 (0.2, 2.5)	<0.0001

*Some data missing

Nicolaou L, *et al. BMJ Global Health* 2020; 5:e003427. doi: 10.1136/bmjgh-2020-003427

eFigure 1. Goodness of fit. Mean and 95% confidence intervals of predicted and observed site-specific and overall head circumference-for-age trajectories with age. Predicted values as obtained from the multivariable linear mixed effects model.

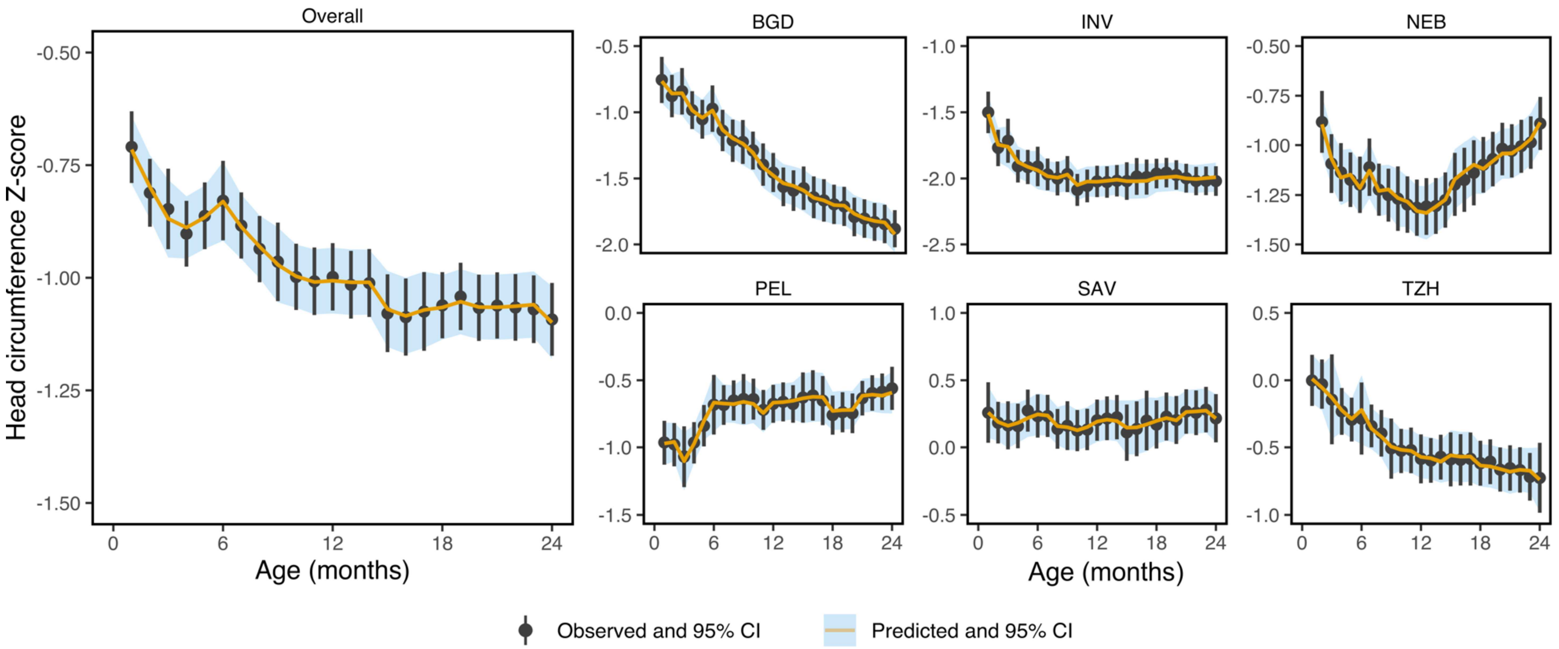

eFigure 2. **Mean and 95% confidence intervals of cognitive, gross motor and language scores as a function of head circumference for age Z-score (HCAZ) and HCAZ slope stratified by site.**

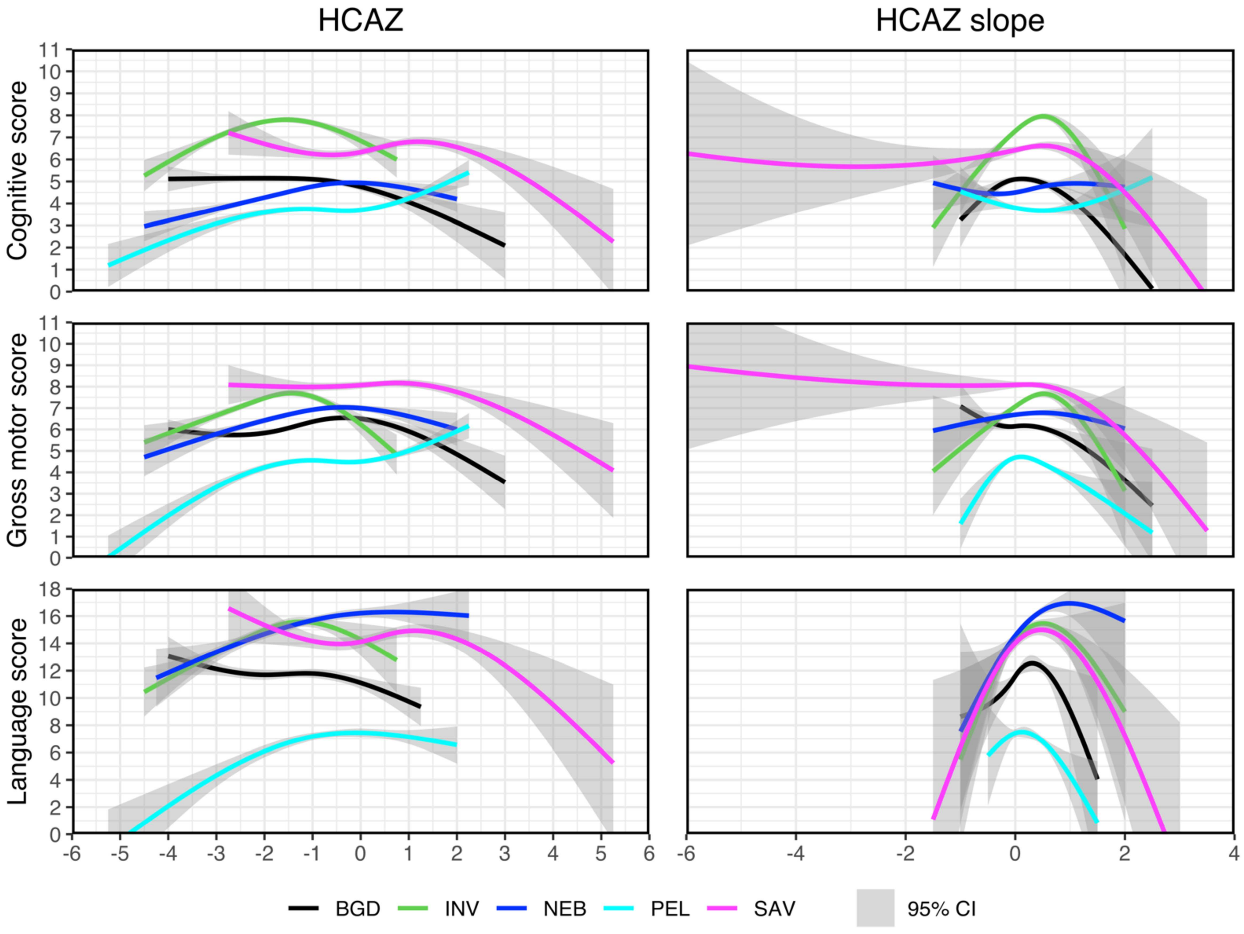

eFigure 3. Head circumference-for-age Z- score vs length-for-age Z-score stratified by site. The ellipses represent the 95% confidence regions for the scatterplot between head circumference-for-age Z- score and length- for-age Z-score each site at 3, 6, 12, and 24 months of age.

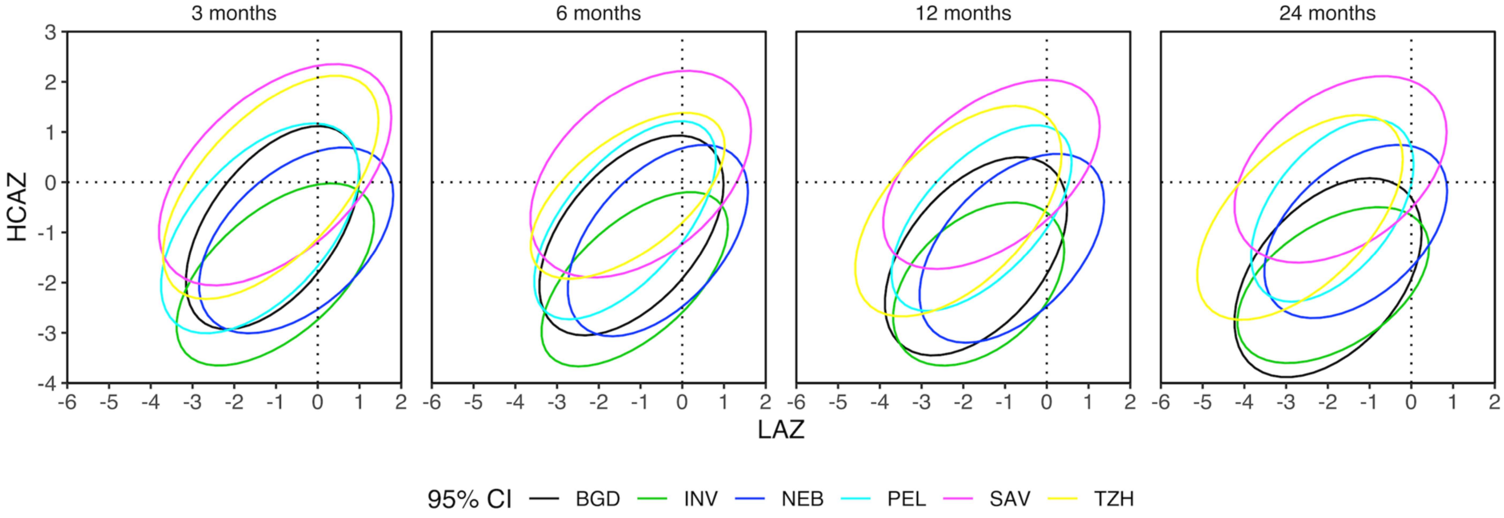

Nicolaou L, *et al. BMJ Global Health* 2020; 5:e003427. doi: 10.1136/bmjgh-2020-003427

eFigure 4. **Overall change in head circumference for age Z-score (HCAZ) at enrollment, 12, and 24 months of age for all risk factors considered.** Estimated mean differences in HCAZ are plotted over ranges in the risk factors between the second, third or fourth quartiles and the first quartile, as obtained from the unadjusted and adjusted linear mixed effects models.

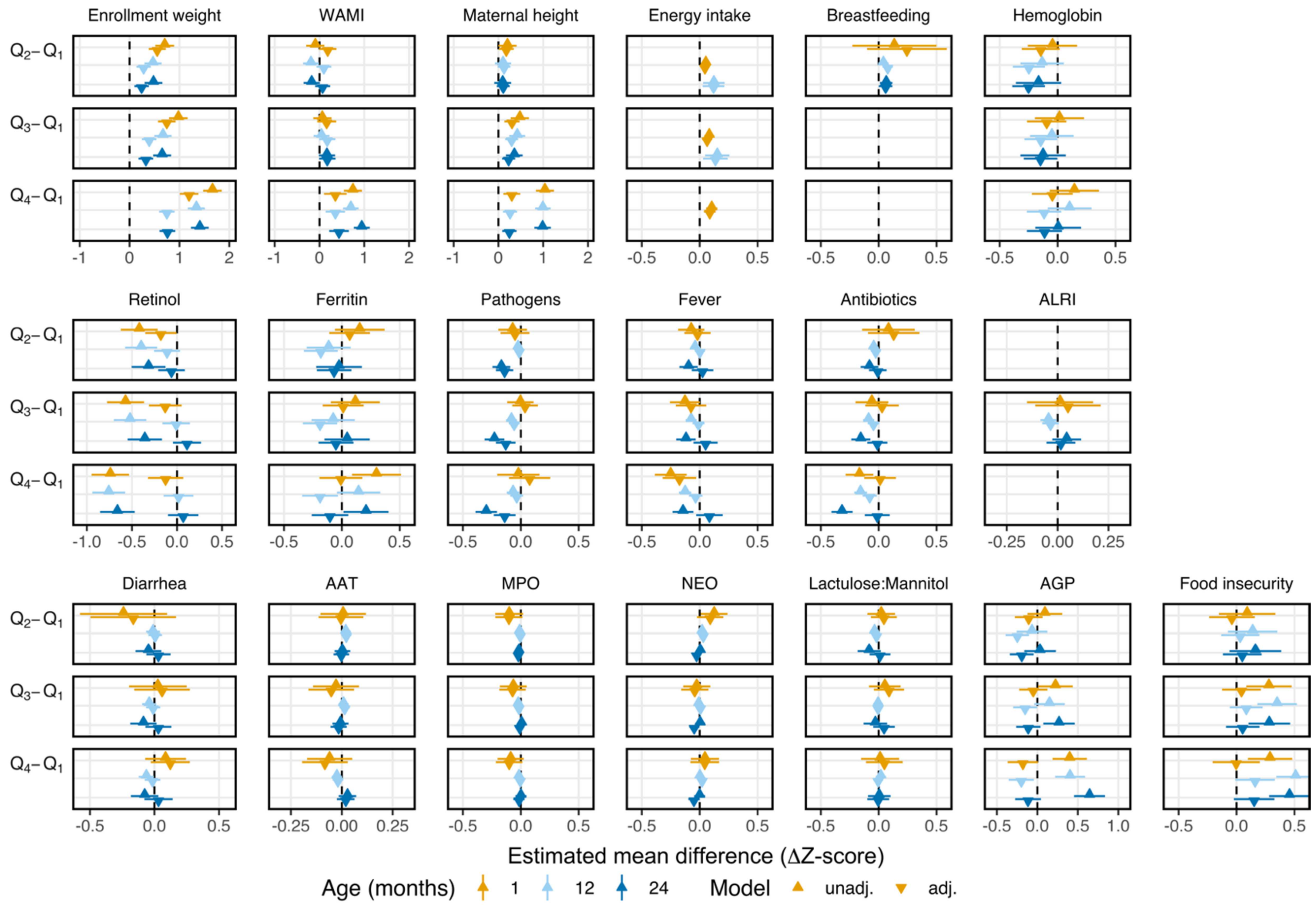

Nicolaou L, *et al. BMJ Global Health* 2020; 5:e003427. doi: 10.1136/bmjgh-2020-003427

eFigure 5. Change in head circumference for age Z-score (HCAZ) by site at enrollment, 12, and 24 months of age for all risk factors considered. Estimated mean differences in HCAZ are plotted over ranges in the risk factors between the second, third or fourth quartiles and the first quartile, as obtained from the unadjusted and adjusted linear mixed effects models. Each symbol represents an individual site.

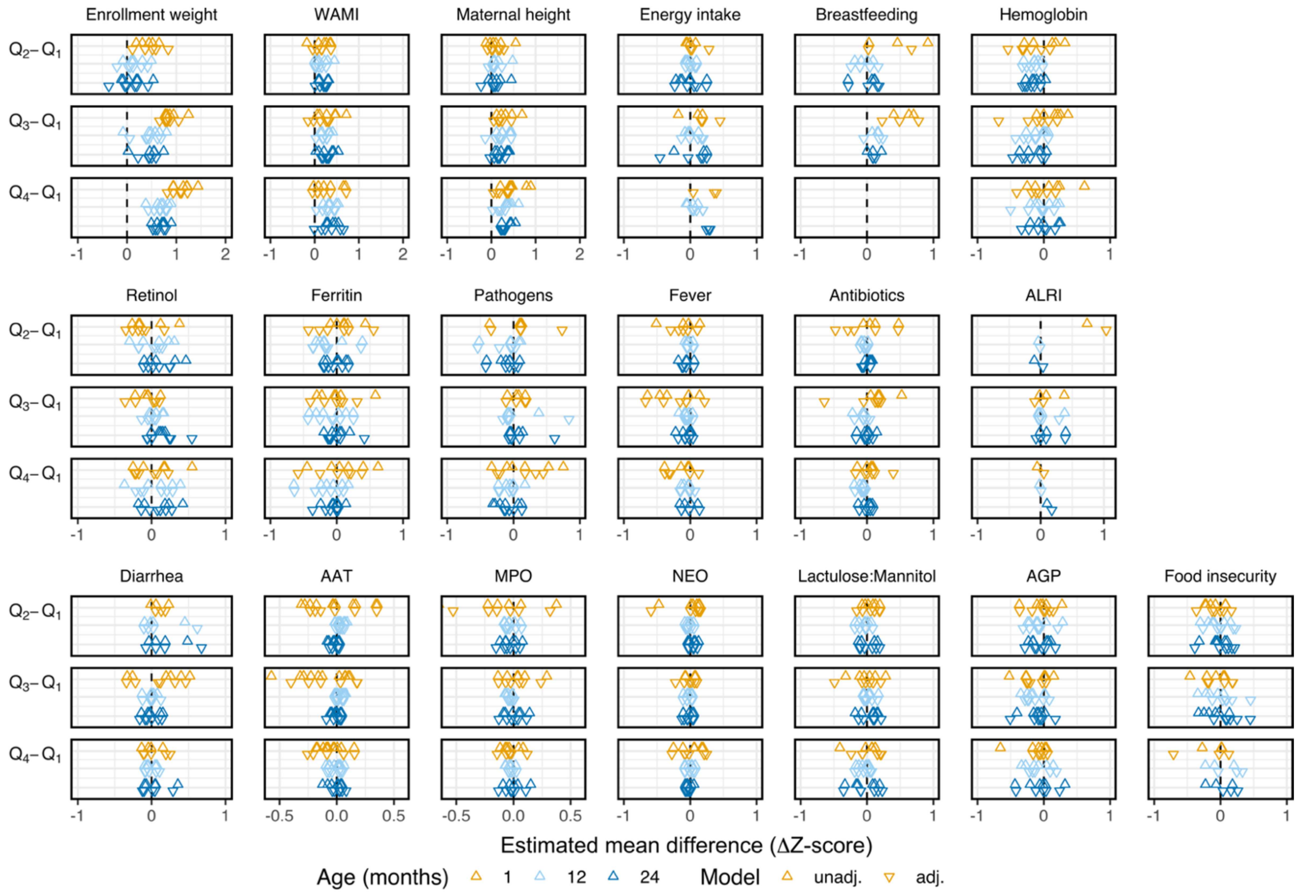

Nicolaou L, *et al. BMJ Global Health* 2020; 5:e003427. doi: 10.1136/bmjgh-2020-003427

eFigure 6. **Change in cognitive, gross motor and language scores with head circumference for age Z-score (HCAZ) and HCAZ slope at 6, 15, and 24 months of age.** Estimated mean differences are plotted over the quartile increases, Q2-Q1 and Q3-Q2, of HCAZ and HCAZ slope as obtained from the unadjusted models and the models adjusted for enrollment weight and site, adjusted for the main factors (enrollment weight, site, HCAZ, maternal height, mean WAMI, mean hemoglobin concentration, pathogen burden, fever episodes), and adjusted for all factors.

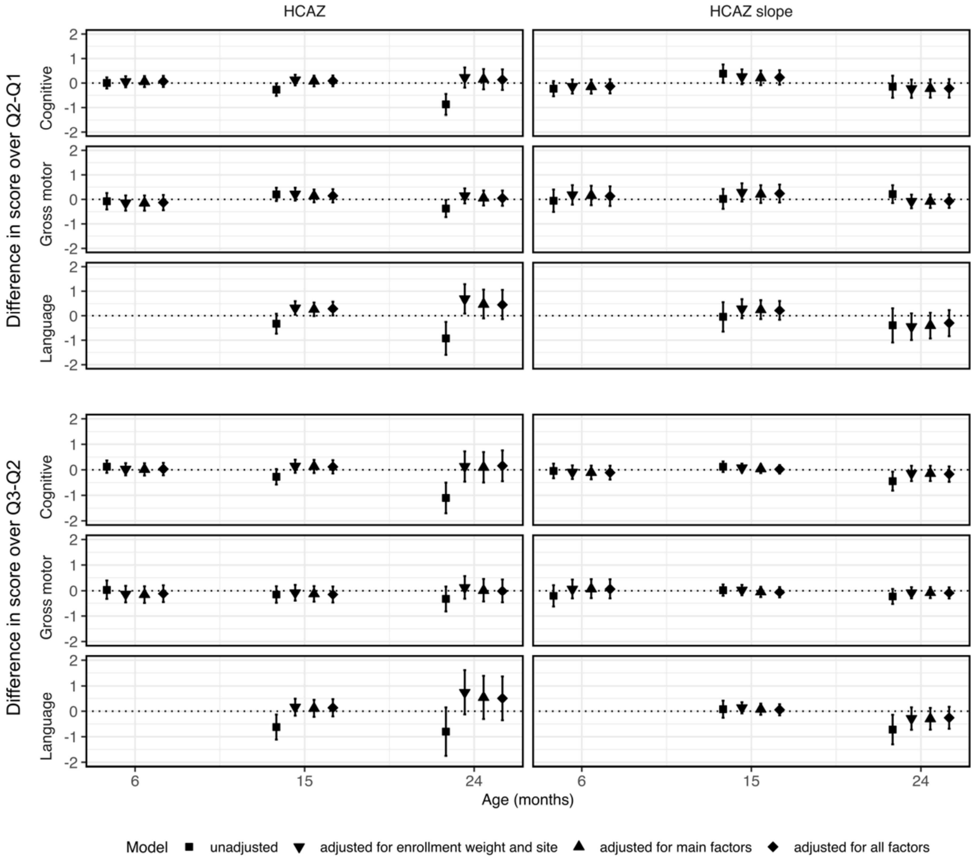

eFigure 7. Change in cognitive, gross motor and language scores with lagged head circumference for age Z-score (HCAZ) at 6, 15, and 24 months of age. Estimated mean differences in the BSID-III test scores are plotted over one standard deviation below the median of HCAZ, taking into account various lags between cognitive function and HCAZ, as obtained from models adjusted for all factors.

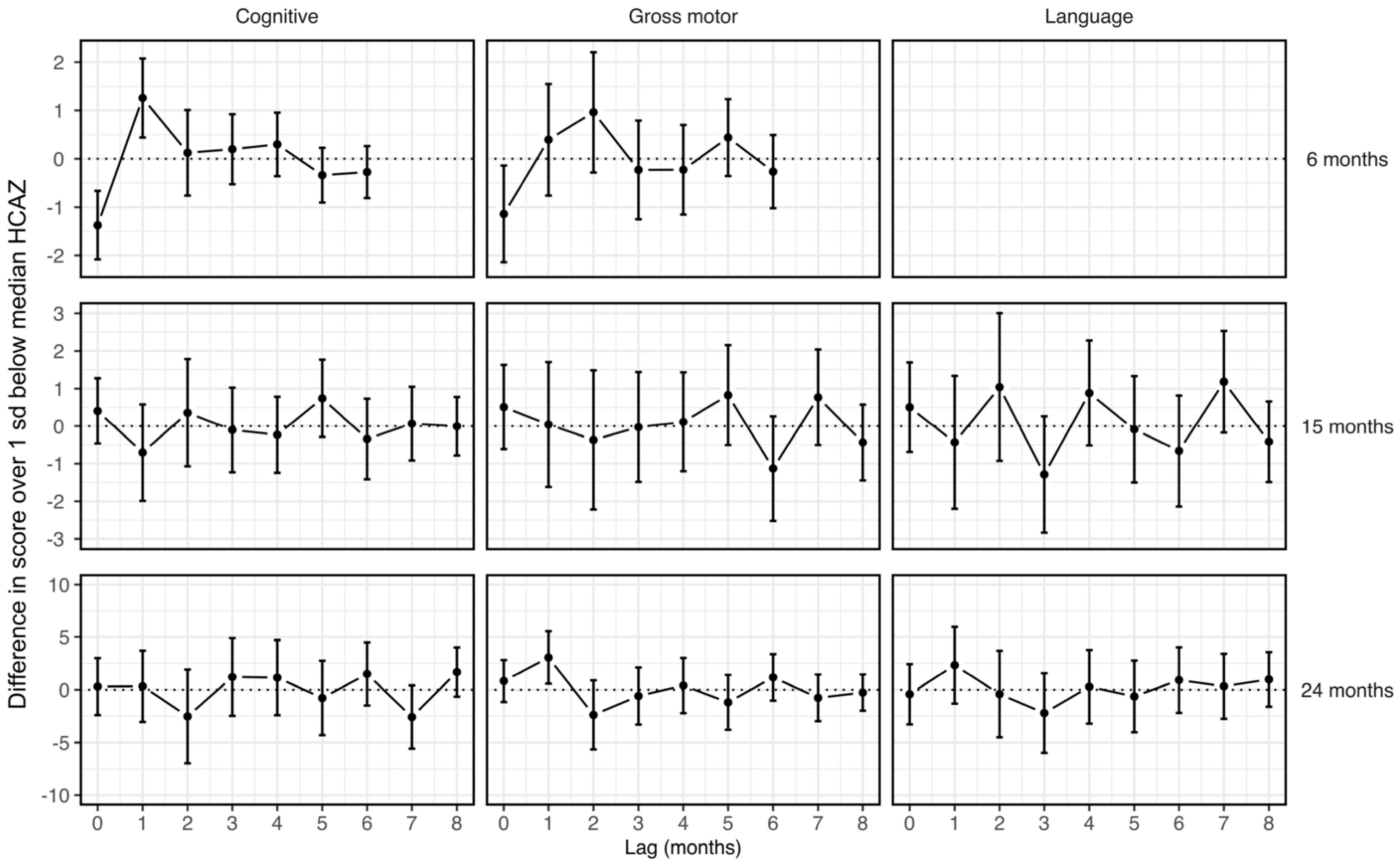

Nicolaou L, *et al. BMJ Global Health* 2020; 5:e003427. doi: 10.1136/bmjgh-2020-003427

eFigure 8. Change in cognitive, gross motor and language scores with lagged head circumference for age Z-score (HCAZ) at 6, 15, and 24 months of age. Estimated mean differences in the BSID-III test scores are plotted over one standard deviation above the median of HCAZ, taking into account various lags between cognitive function and HCAZ, as obtained from models adjusted for all factors.

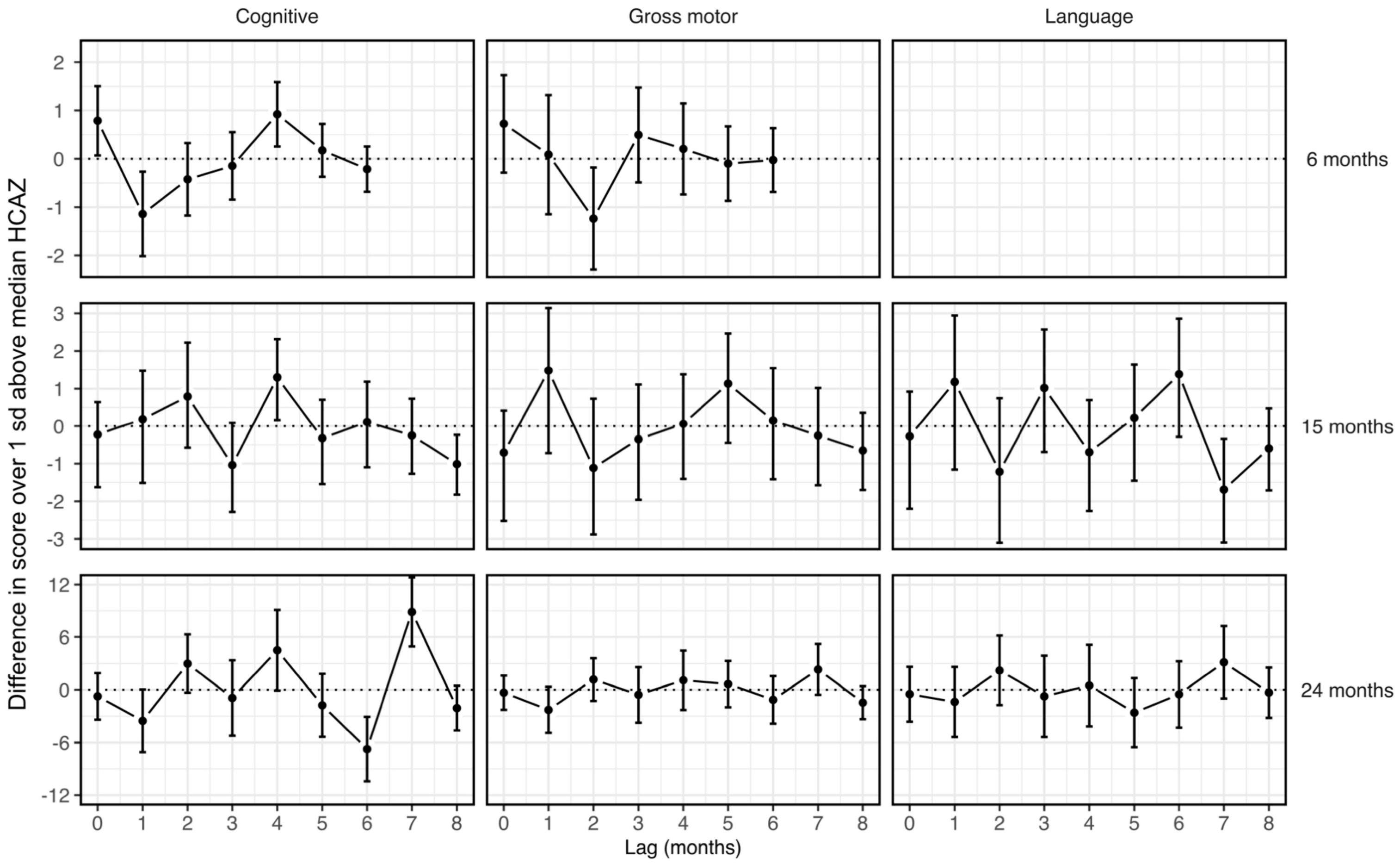

eFigure 9. Mediation of head circumference on cognitive function during early life development. The symbols represent the direct (ADE), head circumference-mediated (ACME) and total effects on cognitive score over the interdecile range of all risk factors considered at 6, 15 and 24 months of age. Error bars represent 95% confidence intervals.

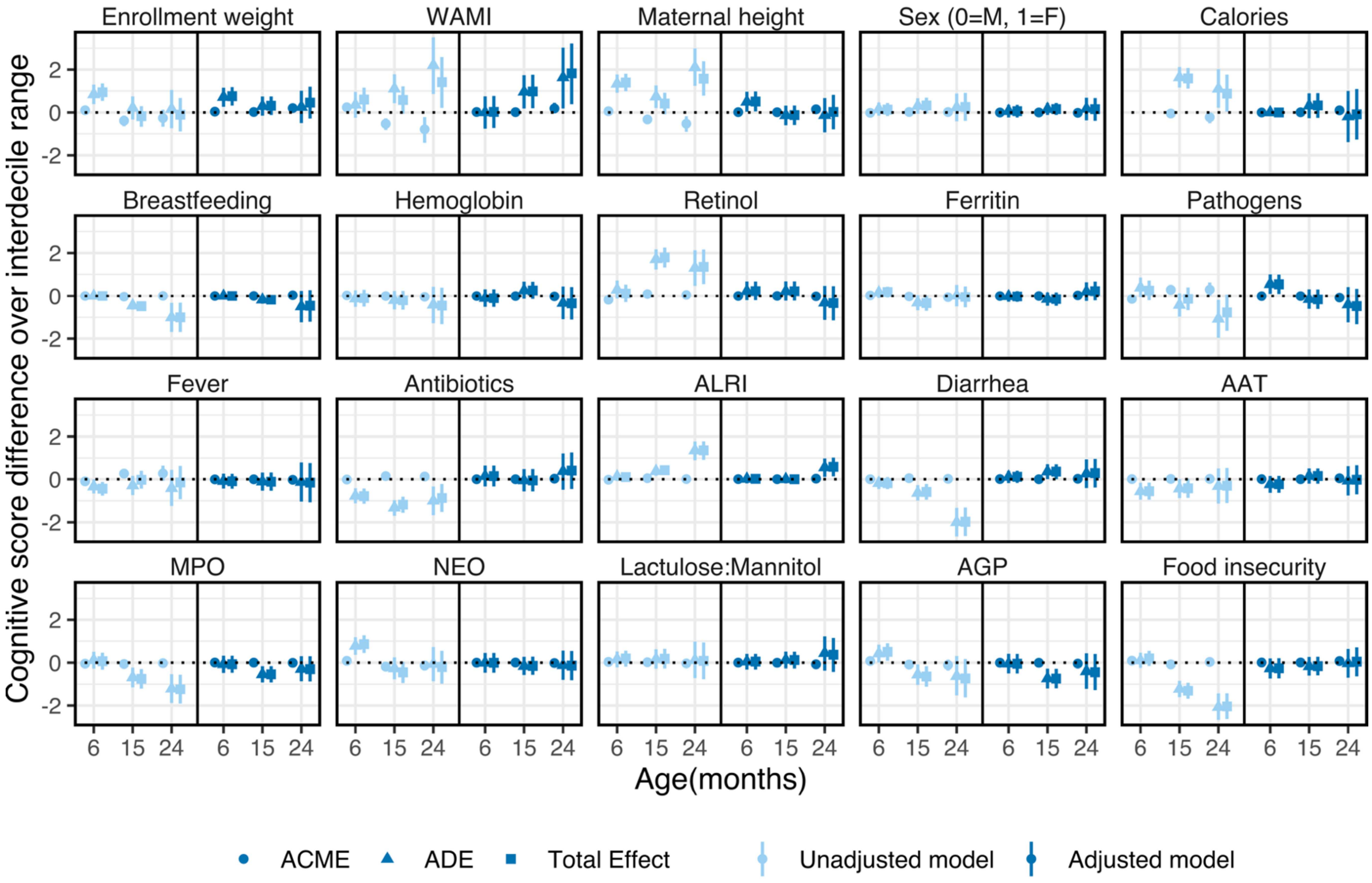

eFigure 10. Mediation of head circumference on gross motor function during early life development. The symbols represent the direct (ADE), head circumference-mediated (ACME) and total effects of various risk factors on gross motor function at 6, 15 and 24 months of age. Error bars represent 95% confidence intervals.

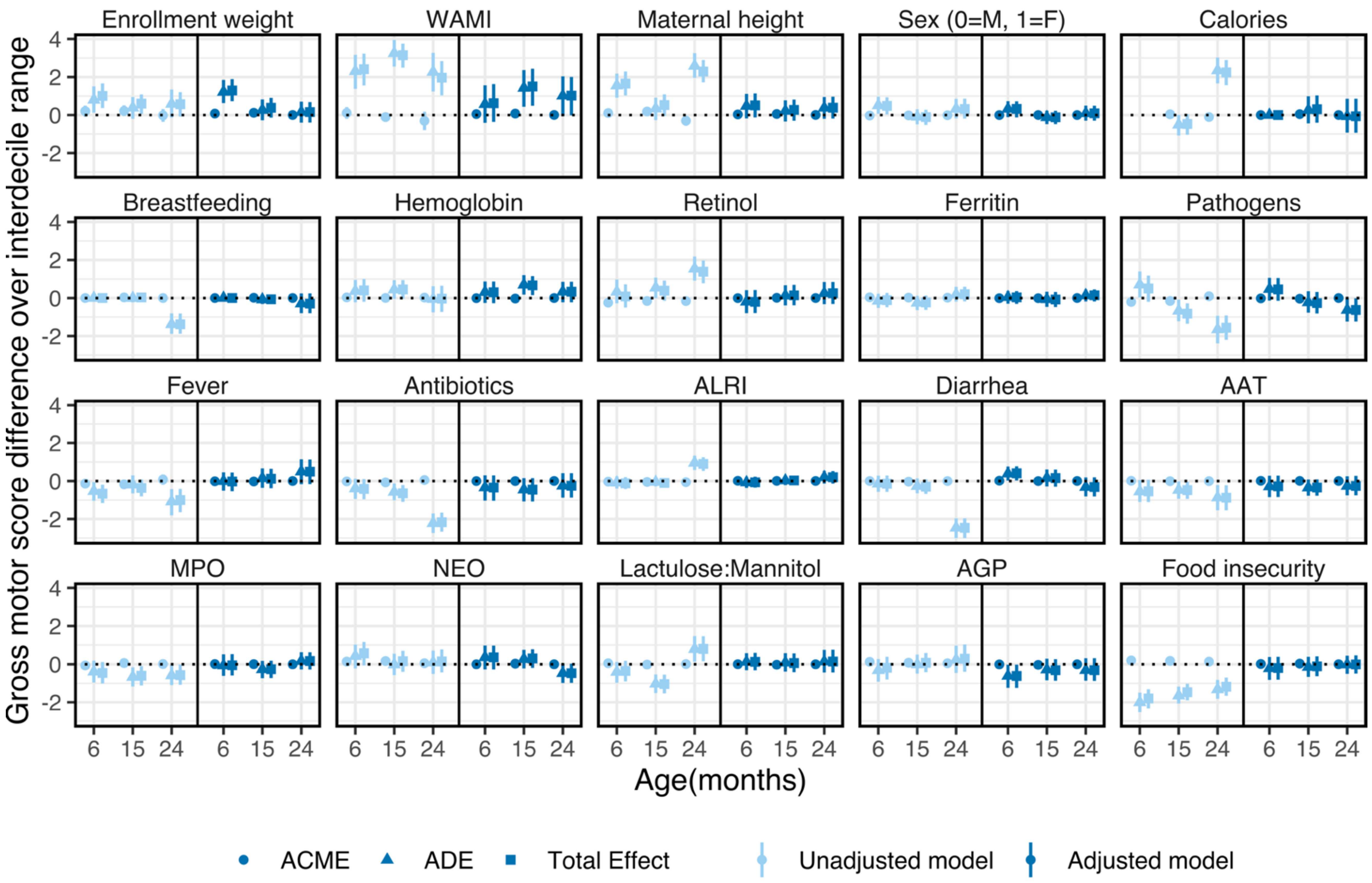

Nicolaou L, *et al. BMJ Global Health* 2020; 5:e003427. doi: 10.1136/bmjgh-2020-003427

eFigure 11. Mediation of head circumference on language skills during early life development. The symbols represent the direct (ADE), head circumference-mediated (ACME) and total effects of various risk factors on language skills at 15 and 24 months of age. Error bars represent 95% confidence intervals.

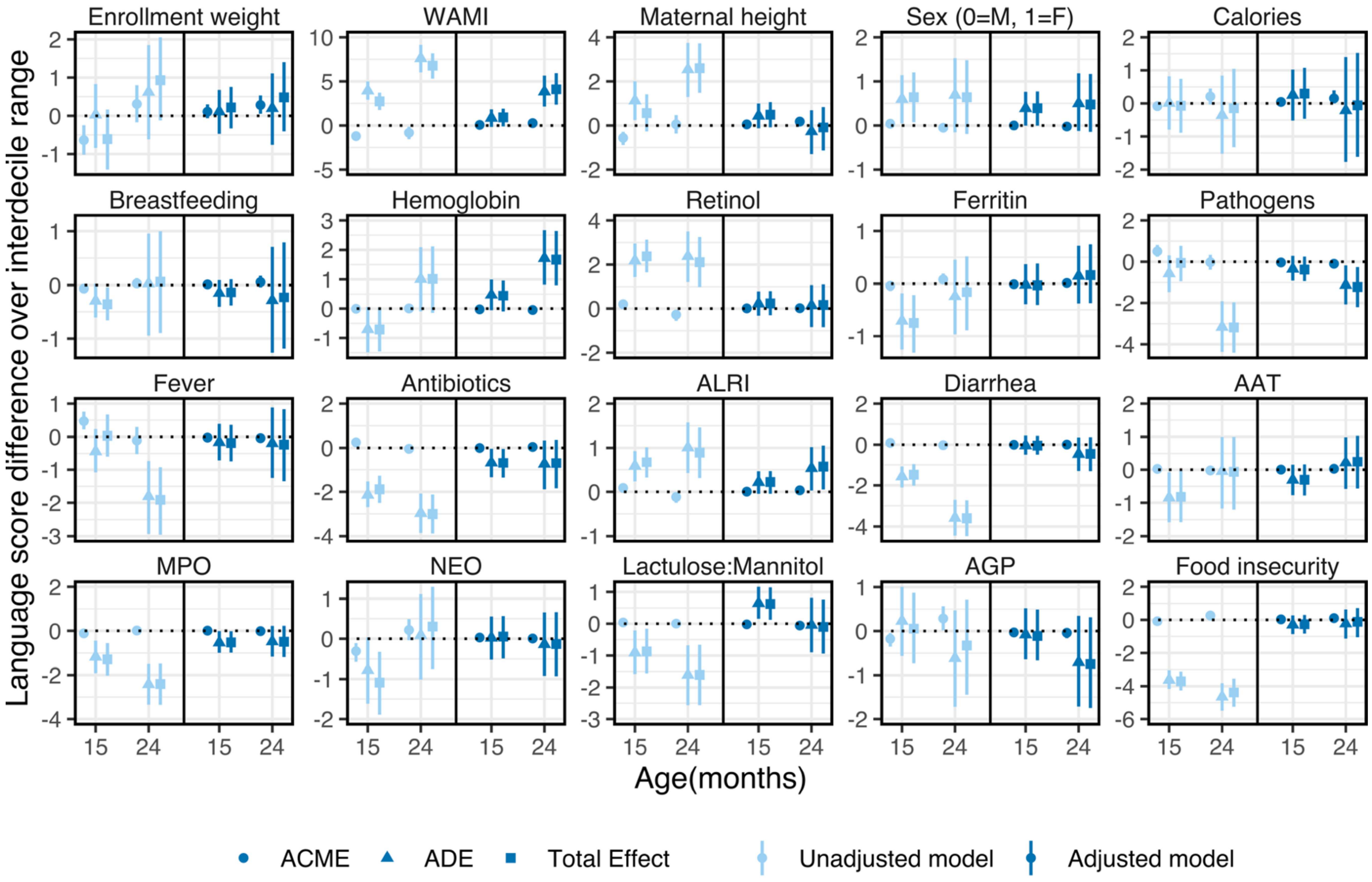

Nicolaou L, *et al. BMJ Global Health* 2020; 5:e003427. doi: 10.1136/bmjgh-2020-003427
